# White Wine—Induced Endothelium-Dependent Vasorelaxation in Sprague-Dawley Rats

**DOI:** 10.3390/antiox11050944

**Published:** 2022-05-11

**Authors:** Zrinka Mihaljević, Toni Kujundžić, Vladimir Jukić, Ana Stupin, Mato Drenjančević, Ines Drenjančević

**Affiliations:** 1 Institute and Department of Physiology and Immunology, Faculty of Medicine Osijek, Josip Juraj Strossmayer University of Osijek, J. Huttlera 4, 31000 Osijek, Croatia; zrinka.mihaljevic@mefos.hr (Z.M.); ana.stupin@mefos.hr (A.S.); 2 Scientific Centre of Excellence for Personalized Health Care, Josip Juraj Strossmayer University of Osijek, Trg Sv. Trojstva 3, 31000 Osijek, Croatia; 3 Department of Fruit Growing, Viticulture and Enology, Faculty of Agrobiotechnical Sciences Osijek, Josip Juraj Strossmayer University of Osijek, Vladimira Preloga 1, 31000 Osijek, Croatia; toni.kujundzic@fazos.hr (T.K.); vladimir.jukic@fazos.hr (V.J.)

**Keywords:** vasodilation, wine, Sprague-Dawley rat, antioxidants, fermentation

## Abstract

The vasodilatory activity and polyphenolic content of commercially available white wine is low compared to red wines. This study assessed the vasodilator potential of white wines produced by four different fermentation processes: (1) white wine produced by the standard procedure; (2) grapes left to macerate completely for 30 days; (3) grapes left to macerate up to half of unfermented sugar; and (4) wine produced by cooling the must. All tested wine samples were analyzed for their phenolic content, antioxidant capacity, and ethanol content. Vasodilation was examined in the norepinephrine pre-contracted isolated rat aortas of male Sprague-Dawley rats randomly exposed to cumulative concentrations (0.1‰ to 8‰ final dilutions in organ baths) of each of the tested wine samples with or without quercetin and/or gallic acid supplementation, in the absence/presence of NOS inhibitor L-NAME. Standard procedure and the procedure involving must cooling gives wine with lower phenolic content, antioxidant capacity, and lower vasodilator potential, respectively. L-NAME inhibited vasodilation to all wine samples. Quercetin with or without gallic acid supplementation restored vasodilation. Results show that vasodilation to white wine is NO-dependent and suggest the possibility of increasing the antioxidant capacity and vasodilatory potential of white wine using different production procedures, depending on quercetin content.

## 1. Introduction

Metabolic syndrome, as well as cardiovascular diseases (e.g., diabetes mellitus DM, obesity, hypertension, and hyperlipidemia), underlie the dysfunction of the endothelium [1,2]. Endothelial dysfunction is primarily characterized by a decrease in the bioavailability of nitric oxide, which acts as a major factor in the relaxation of vascular smooth muscle with consequent dilatation [3,4,5]. A number of studies, both in vitro and in vivo in animal models of diabetes mellitus, have shown that the use of polyphenolic flavonoid compounds (e.g., quercetin) can reduce endothelial dysfunction and also improve endothelium-dependent dilatation through a protective effect on production and nitric oxide (NO) bioavailability [6,7,8,9]. 

In addition to the direct endothelium-dependent vasodilatory effect of polyphenols from red wine [10], it has been shown that these polyphenols can have antioxidant and protective effects on the endothelium in the prevention of various cardiovascular diseases. On the contrary, white wines’ polyphenols content and vasorelaxing effect, compared to red wines, are mainly reduced [11]. Polyphenols intermediate a positive effect on the endothelium–beside an antioxidant effect–by inhibiting the endothelial production of vasoconstrictors (e.g., endothelin-1) but also by promoting the production of vasodilator metabolites (e.g., NO and other endothelium derived hyperpolarizing factor (EDHF)) [12].

Dietary polyphenols, e.g., quercetin, are present in substantial amount in various vegetables, fruits, vines, and tea, and have beneficial effects on the vessel wall [13,14,15]. They delay atherosclerotic processes [16] and reduce hypertension [17]. Quercetin was found to be a reducing co-substrate of the second step in the cyclooxygenase (COX) enzymatic reaction [18], thus affecting endogenous prostanoid production. Quercetin is a plant pigment also found in grapes that acts as a powerful antioxidant. High concentrations of quercetin are found in red wine and tea. Quercetins’ glucuronide and sulfate conjugates appear in blood plasma [19]. These metabolites are cleaved in the peripheral tissues in situ, including the vessel wall [20]. Numerous beneficial effects of quercetin and other flavonoids on the cardiovascular system are known [17,21,22,23,24]. One of the most important properties of quercetin is the ability to modulate inflammation by inhibiting inflammatory COX enzymes and lipoxygenase, thereby reducing the inflammatory mediators prostaglandins and leukotrienes [18]. Interestingly, in a study by García-Mediavilla V et al. [25], quercetin was shown to have the ability to reduce mRNA levels of inducible nitric oxide synthase (iNOS), cyclooxygenase-2 (COX-2), and expression of C-reactive protein (CRP), and bring about reductions in iNOS, COX-2, and CRP levels at low and medium concentrations in human hepatocytes. This study suggests that modulation of iNOS, COX-2, and CRP by quercetin contributes to anti-inflammatory effects through mechanisms involving blockade of nuclear factor kappa B (NF-κB) activation [25].

Based on previous research [10,26,27] and the fact that white wines can be produced from red grapes, it can be concluded that the observed lower effectiveness of white wines on vascular relaxation and generally reduced impact of white wines on the cardiovascular system and human health can be influenced by the wine production process, and could be improved by better extraction of polyphenols as vasoactive substances and other components from grapes by changing wine-making production processes. New wine producing technological processes were designed and resulted in a so called “orange wine”–a fourth type of wine after red, white, and rosé that can be described as a white wine produced by the red wine production technique. Orange wine products are made from white grape varieties, as in the case of white wine, but that is the only thing they have in common. The taste often leaves a distinctive texture, body, and range of tannins on the palate, just like red wines, and has the fruit and mineral content of white wine [28].

The present study assessed the vasodilator potential (in vitro in aortic rings of healthy male Sprague-Dawley rats) of white wines produced by four different fermentation processes and investigated the role of NO in these effects. Thus, the purpose of this study was to test the hypothesis that various wine production procedures leads to increased phenolic content, which underlies improved white wine-induced endothelium-dependent vasorelaxation mediated by phenols and flavonoids, in contrast to the effects of standard white wine production procedure. The experiments conducted in the present study were designed to: (1) test the effect of white wine samples on vascular endothelium-dependent reactivity in Sprague-Dawley rats; (2) assess effect of gallic acid and quercetin supplementation in wine on vascular reactivity; (3) analyze wine content regards to phenols, flavonoids, alcohol strength, acids, and other compounds; and (4) determine the antioxidative capacity of wine samples produced by different technological procedures.

## 2. Materials and Methods

### 2.1. Wine Samples

Wines were made from hand-harvested grapes of Graševina (VIVC-No 13217) in 2020 at the Faculty of Agrobiotechnical Sciences Osijek, Vine and Wine Experimental Station Mandićevac (lat. 45.368428, lon. 18.246395, elevation 200 m) in the eastern continental region of Croatia, the subregion of Slavonia, and among the winegrowing district of Đakovo. The vineyard was planted in 2013, situated at the soil transitioning from the luvisol to the stagnic luvisol with south-facing exposure and a W→E inclination of 9.8%. The soil’s chemical properties resulted in an acid reaction. A single Guyot training system was applied; one long cane with 10 buds and one replacement spur with two buds. The distance between the rows was 2.2 m and 0.8 m within rows, resulting in 5681 vines/ha. After harvest, grapes were subjected to different treatments. In all cases, 25 mg SO_2_/L was added to must or mush and was than inoculated with 250 mg/L Lalvin QA23 (*Saccharomyces cerevisiae*). The standard procedure followed a classic methodology requiring must to be separated from the hard parts of the grapes, without maceration, within 12 h of settling and with fermentation at 25 °C (Wine samples 10–12, group WW_10–12_). The second group of wine was fermented on the skin during 30 days at 25 °C (Wine samples 1–3, WW_1–3_ group). The third wine spent 15 days on the skins, and the rest of the fermentation was conducted without skin (Wine samples 4–6, WW_4–6_ group). The fourth wine of Graševina spent 24 h on the skins at 4 °C (Wine samples 7–9, WW_7–9_ group). After that, must was separated from skin and inoculated with yeast, as described above, and fermented at 25 °C. The finished wines were sulfited with 25 mg/L of SO_2_. When the fermentation ended three months later, the wines were bottled and stored at 12 °C until required for analysis.

### 2.2. Laboratory Animals

The animals were bred and housed at the animal care facility of the Faculty of Medicine Osijek. All experimental procedures conformed to the European Convention for the Protection of Vertebrate Animals used for Experimental and other Scientific Purposes’ (Council of Europe No 123, Strasbourg 1985) European Guidelines for the Care and Use of Laboratory Animals (directive 86/609) and were approved by the Ethical Committee of Faculty of Medicine Osijek, Croatia (#2158-61-07-20-174 and 2158-61-07-20-190, 16 December 2020). A total of 40 male Sprague-Dawley (SD) rats (age 9–12 weeks) were used in this study. Rats were housed in a temperature- (21 °C–23 °C), humidity-, and light-controlled room with free access to tap water, and fed ad libitum with a commercially prepared pellet diet (Mucedola, Settimo Milanese, MI, Italy).

### 2.3. Experiments on Isolated Aortic Rings

The isolated aortic ring experiments were conducted according to the well-established protocol in our laboratory [29,30,31,32,33]. After anesthesia with 75 mg/kg ketamine and 0.5 mg/kg midazolam, thoracotomy was made and the thoracic aorta was isolated, cut to a 3–4 mm ring width, and then placed in an organic pool (10 mL volume) with Krebs-Henseleit’s solution (solution composition in mmol/L: 120 NaCl, 4.8 KCl, 1.2 KH_2_PO_4_, 2.5 CaCl_2_, 1.2 MgSO_4_, 25.5 NaHCO_3_, 10 glucose and 0.02 EDTA) continuously heated and oxygenated (t = 37 °C, pH = 7.4). After rinsing and stabilization for an hour, the endothelial preservation and maximum contraction tests (induced with 60 mM KCl + 10^−7^ M noradrenaline) were conducted, followed by randomized exposition of NA (noradrenaline, 10^−7^ M final concentration) precontracted rings to cumulative concentrations (0.1‰ to 8‰ final dilutions in organ baths) of each of the tested wine samples in the absence/presence of NOS inhibitor L-NAME (3 × 10^−4^ M). The presence of functional endothelium was assessed in all preparations by determining the ability of acetylcholine (10^−5^ M) to induce more than 50% relaxation of rings precontracted with NA (10^−7^ M). There were four groups of three white wine samples numbered 1–12, in which every group presented a different wine-making procedure, as described above. The researcher performing in vitro aortic ring experiments was ignorant of the type of wine or content of potential antioxidants in the samples. Since there was a difference in alcoholic strength between groups of wine samples, the effect of ethanol in similar concentrations as in wine samples on vascular tension was tested by exposition of NA (noradrenaline, 10–7 M final concentration) precontracted rings to ethanol (EtOH) cumulative concentrations (0.1‰ to 8‰ final dilutions in organ baths).

### 2.4. Gallic Acid and Quercetin Supplementation

After determination of the total phenolic and biochemical composition of wine samples, a second set of the experiments on isolated aortic rings was conducted using wine samples 7–12 with supplement of gallic acid only, quercetin only, and both together, to the mean concentration levels measured in wine samples 1–6, 1 mg/g fresh weight (FW) and 25 mg/g FW, respectively, and by using the water solution of gallic acid and/or quercetin (DMSO and Krebs solution in 1:4 ratio) in the same concentration. Aortic rings preparation was the same as described above. NA (10^−7^ M) precontracted aortic rings were exposed to cumulative concentrations (0.1‰ to 8‰ final dilutions in organ baths) of freshly prepared solutions of gallic acid, quercetin, or both together.

### 2.5. Determination of Phenol Content

For spectrophotometric determination of total phenols by Folin-Ciocalteau [34], gallic acid is used as a standard, and the absorbance of ethanolic extracts is measured at 765 nm on a Varian Cary 50 UV-Vis spectrophotometer (Agilent Technologies, Inc., Santa Clara, CA, USA), as previously described.

### 2.6. Determination of Flavonoid Content

From ethanol samples (prepared for phenols), flavonoids are measured spectrophotometrically at 415 nm wavelength on a Varian Cary 50 UV-Vis spectrophotometer (Agilent Technologies, Inc., Santa Clara, CA, USA), according to Ordonez et al. [35]. Quercetin was used as a standard.

### 2.7. Determination of Antioxidant Activity Using the DPPH, TBARS and FRAP Methods

DPPH is determined according to Brand-Williams [36]. The absorbance is measured at 517 nm on a Varian Cary 50 UV-Vis spectrophotometer (Agilent Technologies, Inc., Santa Clara, CA, USA). Vitamin C solution is used as a standard. The concentration of lipid peroxidation products was determined as the amount of substances that react with thiobarbituric acid reactive substances (TBARS) [37]. Spectrophotometric readings were carried out at 532 and 600 nm on a Varian Cary 50 UV-Vis spectrophotometer. As for the FRAP method, as previously described [38], absorbance from ethanol extracts was measured at 593 nm on a Varian Cary 50 UV-Vis spectrophotometer (Agilent Technologies, Inc., Santa Clara, CA, USA).

### 2.8. Statistical Analysis

All results are expressed as an arithmetic mean ± standard deviation (SD). *p* < 0.05 was considered statistically significant. White wine-induced relaxation (WWIR) is expressed as a percentage of the maximum contraction. The aortic rings’ responses to white wine samples were analyzed by Two-way ANOVA with a post hoc Bonferoni test, otherwise ordinary One-way ANOVA test or Kruskal-Wallis test, followed by the Holm-Sidak post hoc test, respectively. SigmaPlot v.12 (Systat Software, Inc., Chicago, IL, USA) and GraphPadPrism, Version 5.00 for Windows, GrafPad Software (San Diego, CA, USA) were used for statistical analysis.

## 3. Results

### 3.1. Experiments in Isolated Aortic Rings

**Figure 1:**
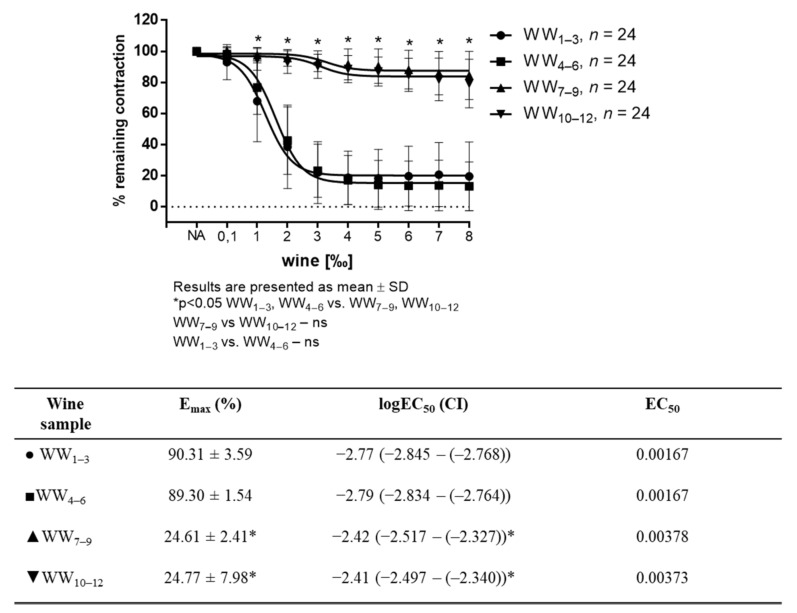
WW-induced relaxation in NA-precontracted (10^−7^ mM) aortic rings (*n* = 8 per wine sample). Tables below graphs presents maximum vasodilation (E_max_) values as mean ± SD values, and the 50% effective concentration (EC_50_) was calculated using nonlinear regression analysis. EC_50_ values are a log of dilution giving 50% of relaxation relative to the sample’s own maximal relaxation (1‰ = 0.001; log 0.001 = −3 and 2‰ = 0.002; log 0.002 = −2.70), with the 95% confidence interval (CI) in parentheses. Results are shown as mean ± SD; * *p* < 0.05 Two-way ANOVA test for vascular tension and One-way ANOVA test for E_max_ and EC_50._.

White wine-induced vasorelaxation (WWIR) was wine-sample- and dose-dependent. Vasorelaxation was significantly different between the groups of differently produced white wines. WWIR in groups of wine samples 1–3 (WW_1–3_) and 4–6 (WW_4–6_) was much stronger than that of 7–9 (WW_7–9_) and 10–12 (WW_10–12_) groups of wine samples, respectively. E_max_ for the WW_1–3_ and WW_4–6_ groups were 90.31% ± 3.59, 89.30 ± 1.54, and for WW_7–9,_ and for groups WW_10–12_ it was 24.61 ± 2.41 and 24.77 ± 7.98, respectively (Figure 1).

**Figure 2:**
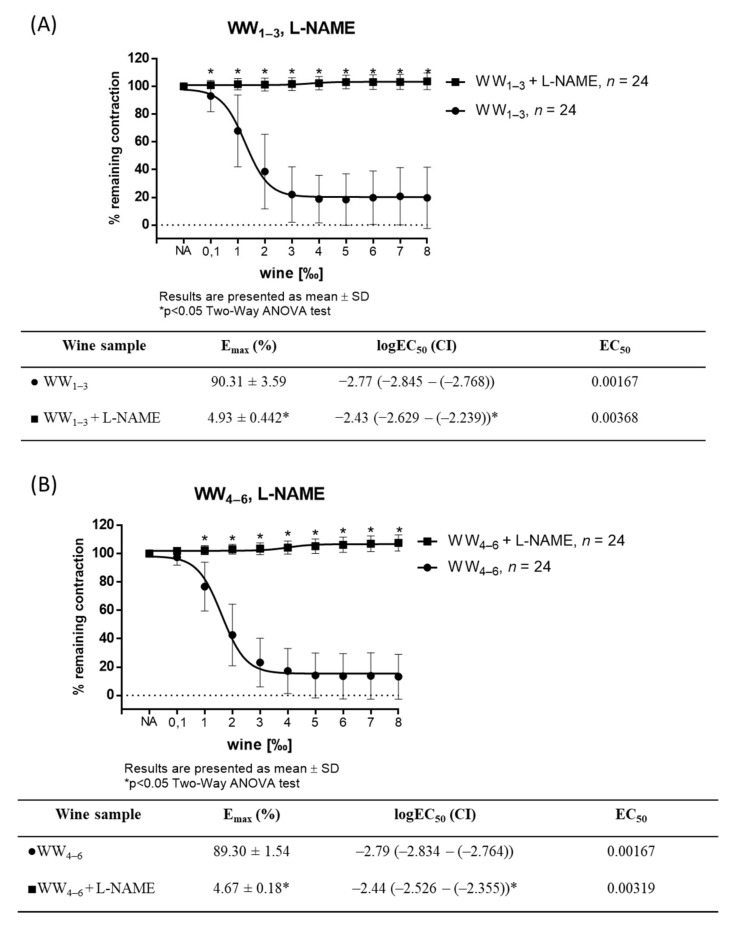

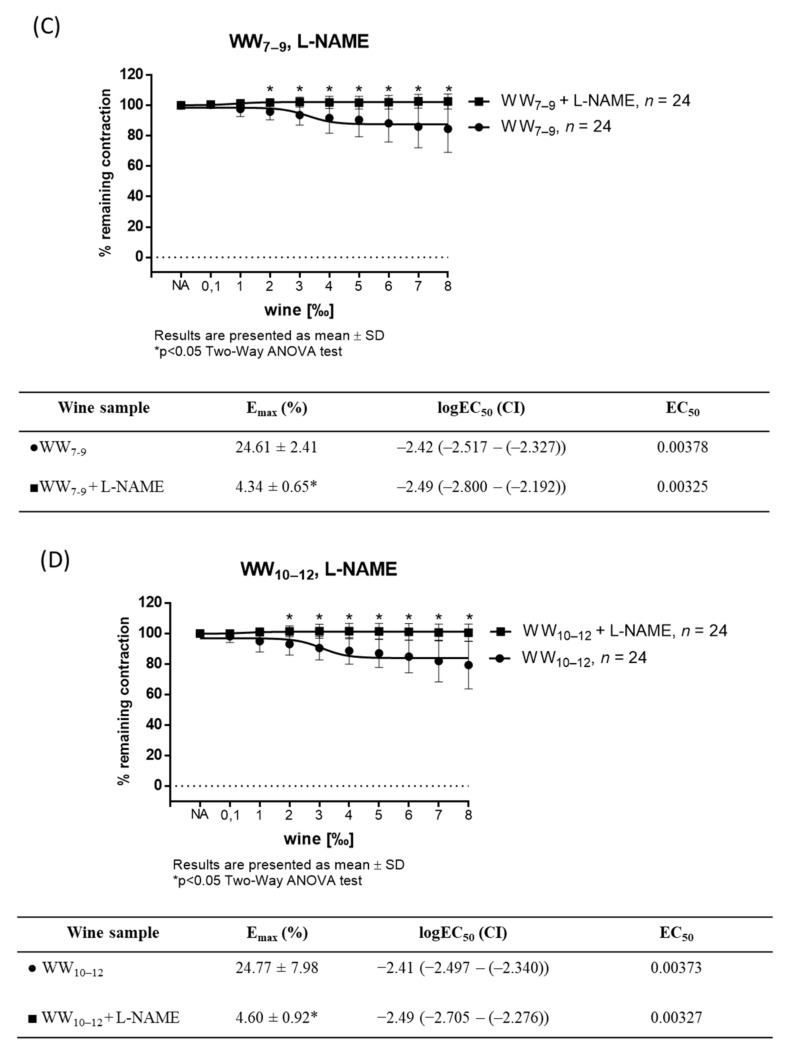

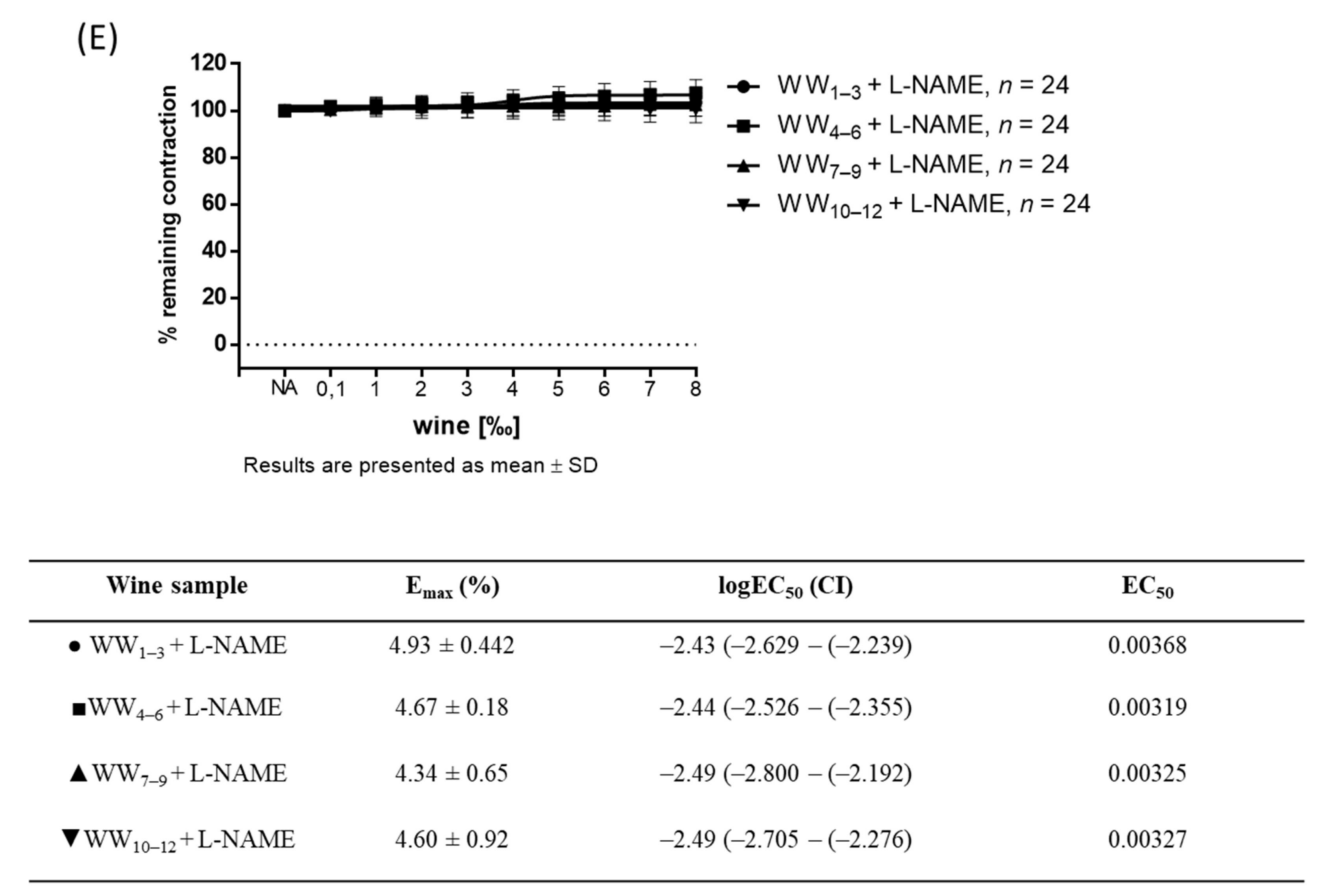
WW-induced relaxation in NA-precontracted (10^−7^ mM) aortic rings with or without 300 mmol/L L-NAME in WW_1–3_ (**A**); WW_4–6_ (**B**); WW_7–9_ (**C**) and WW_10–12_ (**D**) groups (*n* = 8 per wine sample (24 per group)) and between groups (**E**). Tables below graphs presents maximum vasodilation (E_max_) values as mean ± values, and the 50% effective concentration (EC_50_) was calculated using nonlinear regression analysis. EC_50_ values are a log of dilution giving 50% of relaxation relative to the sample’s own maximal relaxation (1‰ = 0.001; log 0.001 = −3 and 2‰ = 0.002; log 0.002 = −2.70), with the 95% confidence interval (CI) in parentheses. Results are shown as mean ± SD; * *p* < 0.05.

Pretreatment of the aortic rings with L-NAME, an inhibitor of NO synthase, completely abolished vasodilator response to all white wine samples (Figure 2A–E).

**Figure 3:**
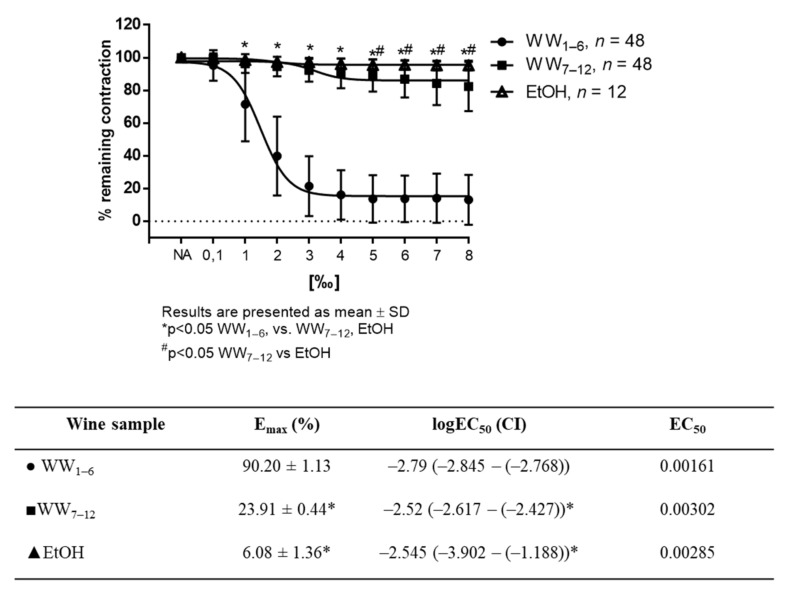
WW- and ethanol induced relaxation in NA-precontracted (10^−7^ mM) aortic rings exposed to cumulative doses (0.1–8‰) of white wine samples or ethanol. Tables below graph presents maximum vasodilation (E_max_) values as mean ± values, and the 50% effective concentration (EC_50_) was calculated using nonlinear regression analysis. EC50 values are a log of dilution giving 50% of relaxation relative to the sample’s own maximal relaxation (1‰ = 0.001; log 0.001 = −3 and 2‰ = 0.002; log 0.002 = −2.70), with the 95% confidence interval (CI) in parentheses. Results are shown as mean ± SD; *^#^ *p* < 0.05 Two-way ANOVA test; LogEC_50_ values (shown in corresponding tables) were compared by a One-Way ANOVA test.

Ethanol in similar concentrations as in tested wine samples had no effect on vascular tension (Figure 3). 

**Figure 4:**
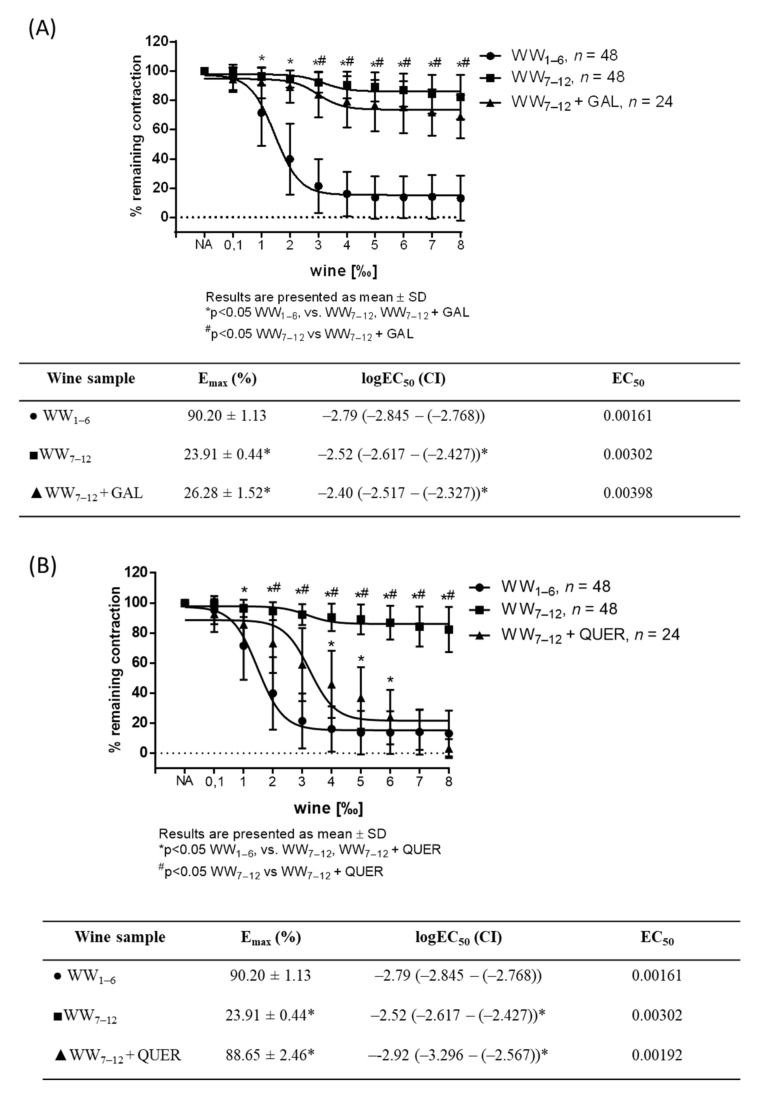

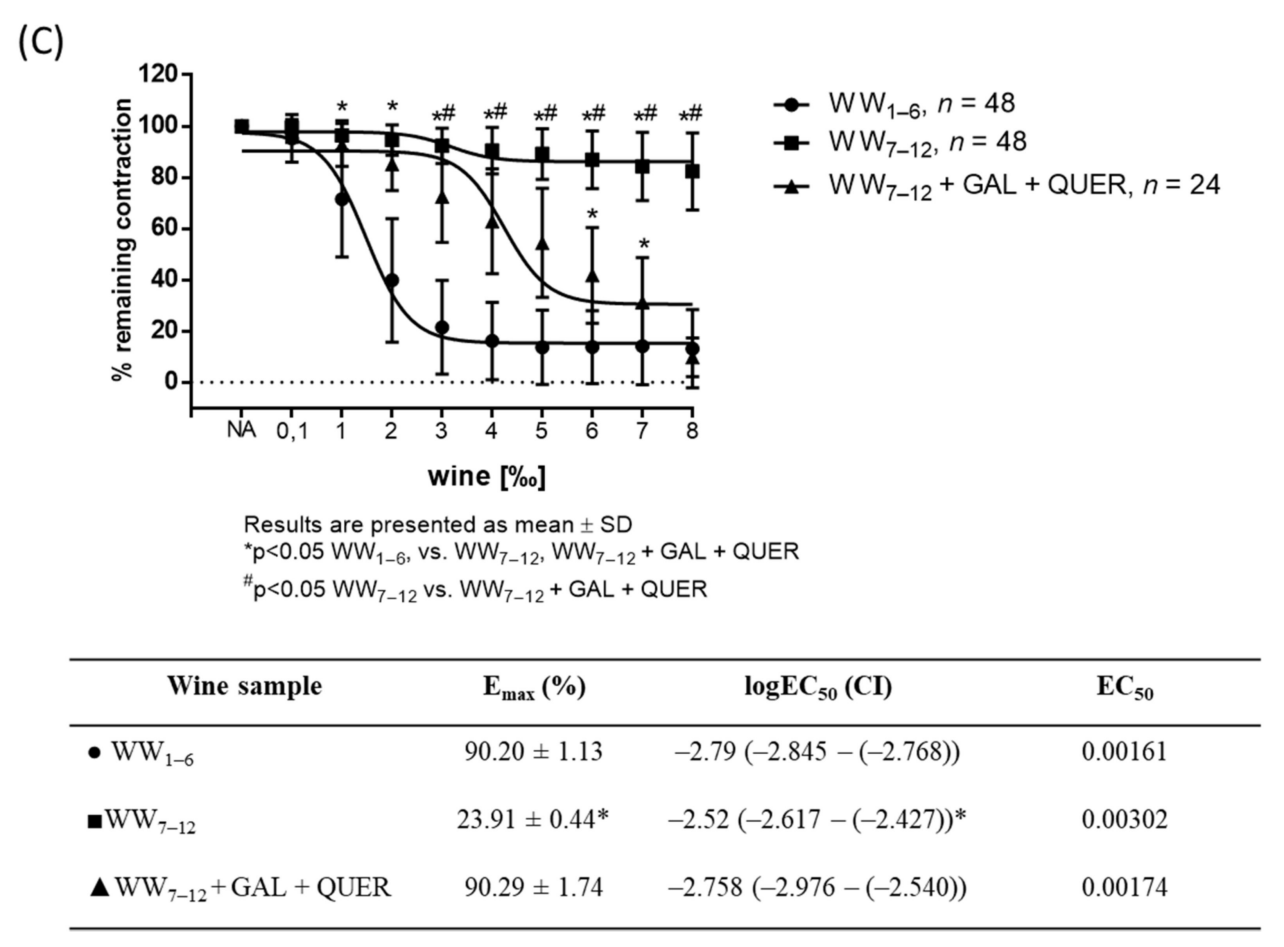
WW-induced relaxation in NA-precontracted (10^−7^ mM) aortic rings with WW_7–12_ wine samples supplemented with gallic acid (**A**), quercetin (**B**) and gallic acid and quercetin together (**C**) compared to non-supplemented wine samples (WW_1–6_ and WW_7–12_; *n* = 8 per wine sample). Tables below graphs presents maximum vasodilation (E_max_) values as mean ± values, and the 50% effective concentration (EC_50_) was calculated using nonlinear regression analysis. EC50 values are a log of dilution giving 50% of relaxation relative to the sample’s own maximal relaxation (1‰ = 0.001; log 0.001 = −3 and 2‰ = 0.002; log 0.002 = −2.70), with the 95% confidence interval (CI) in parentheses. Results are shown as mean ± SD; *^#^ *p* < 0.05.

According to the phenol and flavonoid content (Table 1) of the wine samples, gallic acid and quercetin levels supplemented in 7–12 wine samples to the mean levels measured in 1–3 and 4–6 wine samples showed a restored vasodilator response when solely quercetin or gallic acid and quercetin together were applied in wine samples (Figure 4A–C), or when the quercetin or quercetin with gallic acid in DMSO and Krebs solution in 1:4 ratio in the same concentration were applied (data not shown). 

These results suggest that changed technological wine-making procedures can result in quality white wines richer in phenols and flavonoids that can also have improved benefit to humans observed through increased vasorelaxation.

### 3.2. Wine Content Analysis

Total phenolic content of wine samples is shown in Table 1. Phenol content [mg gallic acid g^−1^ FW] and flavonoid content [μg quercetine g^−1^ FW] in WW_7–9_ and WW_10–12_ groups of wine samples compared to WW_1–3_ and WW_4–6_ groups were significantly decreased. Increased phenolic content is achieved using different technological wine-making process.

Biochemical composition of wine samples is shown in the Table 2. Alcoholic strength [vol %] and free sulfur dioxide [mg/mL] in WW_7–9_ and WW_10–12_ groups of wine samples compared to WW_1–3_ and WW_4–6_ groups were significantly increased, whereas total dry extract [g/L], and pH were significantly decreased. Total sulfur dioxide [mg/L] is increased in WW_10–12_ group compared to the other groups and total acids [g/L] were significantly increased in WW_10–12_ group and in WW_4–6_ compared to other groups, respectively. These values are in standard intervals for white wine quality control observed parameters (legally permitted).

Antioxidant capacity of wine is assessed through FRAP, TBARS and DPPH. FRAP in equivalents Fe (II) [mM g^−1^ FW], and TBARS [nmol g^−1^ FW] were significantly decreased in WW_7–9_ and WW_10–12_ groups of wine samples compared to WW_1–3_ and WW_4–6_ groups, and DPPH 50% EC [mg FW/mL] was significantly increased, respectively. These results suggesting improved antioxidant capacity of WW_1–3_ and WW_4–6_ compared to other two groups of tested wines (Table 3).

## 4. Discussion

The main findings of the present study are as follows: standard white wine production processes results in wine with low vasodilator potential and antioxidative status. These results suggest that impaired vasorelaxation of wine obtained with standard procedures may be influenced by increased oxidative stress parameters, decreased pH values, and lower phenol and flavonoids levels. 

Previous studies that explored differences between red and white wine supported the red wine as more beneficial for human health [10,11,39]. Various study showed that the beneficial effect of red wine comes from phenolic content, which is lower in white wine compared to the red wines [10,40,41]. It has also been shown that red wine consumption has a significantly better effect on the prevention of cardiovascular diseases compared to other alcoholic beverages [39]. In the research of Fitzpatrick et al. [10], it was proved that one of the examined white wines has a vasodilating effect, which is significantly less than the vasodilating effects of red wines. 

Based on these previous studies [10,11,39,40,41], it can be concluded that white wines contain certain vasodilating substances, and they depends on the type of grapes used for wine production and on the procedures prior the fermentation. Higher amounts of phenolic compounds in red wines compared to white wines are derived from the grape skin and other parts (seeds, pulp, and even stems) that are extracted from the wine during maceration as the first stage of alcoholic fermentation [28]. White wines are standardly produced from free-running juice without maceration, and its application in white wine production increases the amount of phenolic compounds and of antioxidant characteristics that are more similar to those of red wines [42]. Consequently, it seems necessary that to be able to produce wines with optimal vasodilator properties, besides to adjust fermentation process, one has to have initial ingredients rich in vasoactive substances, but also use specific preparations. Therefore, technologists are trying to find new ways to produce white wines with preserved phenol levels and to achieve the same beneficial effects in white wines than that of red wines. Procedures used to produce wine samples 1–3 and 4–6 in our study seemed to be more effective in producing wines with increased vasodilatory potential and antioxidant capacity.

Previously, de Oliveira et al. [43] showed the vasorelaxation effect of gallic acid and Li et al. [44] showed quercetin-mediated vasodilation. Based on these findings and the significantly lower levels of both components measured in wine samples 7–12, we have hypothesized that quercetin and gallic acid might be responsible for the relaxant effect of white wine. The hypothesis was partially confirmed with the restored vasodilator response of wine samples with low vasodilator potential to the levels seen in wine samples with high vasodilator potential after supplementation of quercetin only. This is in accordance to the previous studies on antioxidant and vasodilatory effects of phenolic acids, which showed that gallic acid was the least effective direct vasorelaxant and most potent antioxidant [45]. Additionally, both endothelium-dependent and -independent mechanisms of quercetin-induced vasorelaxation have been described, in both in vivo and in vitro experiments [7,8,46]. Our results confirm those previous findings, suggesting quercetin as the most potent vasodilator.

Ha SK et al. [26] showed that the vasorelaxation property of dealcoholized wine depends on the endothelium, due to the endothelial ability to release endothelium-derived relaxing factors (EDRF), which leads to vasorelaxation of vascular smooth muscle and maintenance of vascular tone. The most important EDRFs are nitric oxide (NO), prostacyclin, and endothelial-derived hyperpolarization factors (EDHF) [47]. NO is formed by the metabolism of L-arginine NO by endothelial cell synthetase, and participates in the relaxation of vascular smooth muscle [48]. Vasodilation is the most important property of NO, which in addition has other important properties such as: adhesion and migration of leukocytes into the arterial wall, prevention of platelet aggregation, and inhibition of vascular smooth muscle cell proliferation [49,50,51,52,53,54,55]. Quercetin and tannic acid, compounds present in the cuticle, have been shown to cause endothelium-dependent aortic smooth muscle relaxation, while resveratrol and malvidin have not relaxed aortic rings [10,56]. Our results showed white wine-induced vasodilation was completely abolished by L-NAME, supporting the role of NO in observed vasodilation in response to white wine. This grants further research on vasoactive substances in white wines, their isolation, and characterization.

Although the vasodilator effect of wine consumption is preferred and considered as vasoprotective for many cardiovascular diseases [57,58], one of the unwished consequence of wine consumption is headache. Some types of wines induce much stronger headaches than others [59]. Sulfur is most commonly used as an antioxidant and antimicrobial preservative during wine production [60]. Legally permitted sulfur dioxide content in white wines without residual sugar (210 mg/L) is higher than the amount permitted in red wine (160 mg/L) (NN 137/2008) [61]. At the same time, red wine is commonly found to be a headache trigger [59]. However, there are no medical research results in literature that confirm that the sulfites cause headaches [59]. Wine-induced headaches may have several triggers–not only sulfites, but also histamine, tyramine, flavonoids, and serotonin [62]. Sulfur content in our study wine samples were under legally permitted values.

In terms of antioxidant capacity of wine, our results are very consistent with previously published results [45,63]. Increasing the phenol content increases the total antioxidant activity. This can be seen from the FRAP values that increase in proportion to the increase in phenol content (highest FRAP and highest phenol in maceration lasting 30 days). Thus, by increasing the duration of maceration, we increased the antioxidant capacity of the wine. Contrarily, the lower the DPPH 50% EC, the higher the antioxidant capacity. Since these values are inversely proportional, the same was confirmed as with the FRAP values, i.e., both methods show increased antioxidant capacity and an increase in maceration duration. It can be seen from the TBARS data that as the duration of maceration increases, there is an increase in the content of lipid peroxidation products. Temperature during fermentation has an effect on lipid metabolism [64]. By lowering the temperature, the content of lipid peroxidation products decreases, because at lower temperatures the catabolic processes of lipid degradation are slowed down [65]. Therefore, our lowest value in the treatment with cooling of the must to 4 °C in relation to room temperature follows this previous study. 

Since our adjusted wine-making process resulted in wines with increased vasodilatory potential and antioxidant capacity related to phenol content, it will be interesting to evaluate in vivo effect and/or effects of these wines in humans. However, at the moment, without conducted experiments, we can only assume potential beneficial effects. A recently published paper by Matute A et al. underlies the need for proper evaluation of methods used for in vivo evaluation of phenol effects of various products [66]. Furthermore, Shahidi et al. showed potentially beneficial effects of phenolic compounds, and that antioxidant properties can be strongly affected not only by their bioaccessibility (digestion and absorption efficiency) but also bioavailability (ratio of active ingredient absorbed and detected in the target site to the total amount of orally ingested drug products) [67].

## 5. Conclusions

White wine-processing technology, which includes maceration, contributes significantly to increasing the content of desirable chemical compounds and antioxidant capacity in wine. Maceration–skin contact with must as a necessary procedure in red wine production–should be carefully dosed in white wine production in order to avoid unwanted flavors and aromas of the wine, where the content of the preferred chemical compounds would be significantly higher than in standard white wine production technology. Depending on enhanced and/or preserved quercetin content, results show vasodilation to white wine as NO-dependent and suggest the possibility of increasing the vasodilatory potential of white wine using different production procedures.

## Figures and Tables

**Table 1:** Phenol and flavonoid content of wine samples.

	WW_1–3_	WW_4–6_	WW_7–9_	WW_10–12_
Phenol content [mg Gallic acids g^−1^ FW]	1.17 ± 0.107 ^#^	0.81 ± 0.017 *	0.27 ± 0.011 *^#^	0.28 ± 0.004 *^#^
Flavonoid content [µg Quercetine g^−1^ FW]	24.07 ± 1.066	25.18 ± 1.258	16.53 ± 1.478 *^#^	14.98 ± 1.434 *^#^

One-Way ANOVA test; * *p* < 0.05 compared to WW_1–3_; ^#^ *p* < 0.05 compared to WW_4–6_.

**Table 2:** Biochemical parameters of wine samples.

	WW_1–3_	WW_4–6_	WW_7–9_	WW_10–12_
Alcoholic strength [vol %]	13.04 ± 0.253	13.13 ± 0.006	13.71 ± 0.045 *^#^	13.72 ± 0.060 *^#^
Total dry extract [g/L]	23.87 ± 0.568	23.57 ± 0.252	18.73 ± 0.416 *^#^	18.07 ± 0.404 *^#^
Free sulfur dioxide [mg/L]	12.69 ± 5.143	11.73 ± 3.844	75.41 ± 4.345 *^#^	53.33 ± 4.052 *^#†^
Total sulfur dioxide [mg/L]	128.00 ± 7.212	131.50 ± 6.841	126.30 ± 4.541	196.40 ± 3.221 *^#^^†^
Volatile acids [g/L]	0.70 ± 0.021	0.65 ± 0.042	0.81 ± 0.026 *^#^	0.66 ± 0.017 ^#^^†^
Total acids [g/L]	4.87 ± 0.130 ^#^	5.28 ± 0.112 *	4.80 ± 0.075 *^#^	6.20 ± 0.173 *^#†^
pH	3.73 ± 0.0265 ^#^	3.60 ± 0.020 *	3.51 ± 0.045 *^#^	2.92 ± 0.030 *^#^^†^

One-Way ANOVA test; * *p* < 0.05 compared to WW_1–3_; ^#^ *p* < 0.05 compared to WW_4–6_; ^†^ *p* < 0.05 compared to WW_7–9._

**Table 3:** Antioxidant capacity of wine samples.

	WW_1–3_	WW_4–6_	WW_7–9_	WW_10–12_
FRAP in equivalents Fe(II) [mM/g FW]	119.30 ± 18.17 ^#^	74.29 ± 3.003 *	32.93 ± 1.283 *^#^	29.56 ± 1.836 *^#^
TBARS [nmol/g FW]	13.84 ± 1.307 ^#^	9.85 ± 1.419 *	1.47 ± 0.014 *^#^	2.79 ± 0.303 *^#^
DPPH 50% EC [mg FW/mL]	10.65 ± 1.791	16.97 ± 0.7992	56.93 ± 9.138 *^#^	59.25 ± 2.415 *^#^

One-Way ANOVA test; * *p* < 0.05 compared to WW_1–3_; ^#^ *p* < 0.05 compared to WW_4–6_.

## Data Availability

The raw data supporting the conclusions of this article will be made available by the authors, without undue reservation.
